# Adults' Performance in an Episodic-Like Memory Task: The Role of Experience

**DOI:** 10.3389/fpsyg.2018.02688

**Published:** 2019-01-21

**Authors:** Gema Martin-Ordas, Cristina M. Atance

**Affiliations:** ^1^Division of Psychology, University of Stirling, Stirling, United Kingdom; ^2^School of Psychology, University of Ottawa, Ottawa, ON, Canada

**Keywords:** episodic memory, episodic-like memory, temporal information, adults, depletion paradigms

## Abstract

Episodic memory is the ability to consciously recollect personal past events. This type of memory has been tested in non-human animals by using depletion paradigms that assess whether they can remember the “what,” “where,” and “when” (i.e., how long ago) of a past event. An important limitation of these behavioral paradigms is that they do not clearly identify the cognitive mechanisms (e.g., episodic memory, semantic memory) that underlie task success. Testing adult humans in a depletion paradigm will help to shed light on this issue. In two experiments, we presented university undergraduates with a depletion paradigm which involved choosing one of two food snacks—a preferred but perishable food and a less preferred but non-perishable food–either after a short or a long interval. Whereas, in Experiment 1, participants were asked to *imagine* the time between hiding the food items and choosing one of them; in Experiment 2 participants *experienced* the time elapsed between hiding the food items and choosing one of them. In addition, in Experiment 2 participants were presented with 2 trials which allowed us to investigate the role of previous experience in depletion paradigms. Results across both experiments showed that participants chose the preferred and perishable food (popsicle) after the short interval but did not choose the less preferred and non-perishable food (raisins) after the long interval. Crucially, in Experiment 2 experiencing the melted popsicle in Trial l improved participants' performance in Trial 2. We discuss our results in the context of how previous experience affects performance in depletion tasks. We also argue that variations in performance on “episodic-like memory” tasks may be due to different definitions and assessment criteria of the “when” component.

## Introduction

Episodic memory is a form of declarative memory that allows people to recall personally experienced events (Tulving, 1983). Importantly, episodic recollection is entwined with a particular phenomenological experience that allows a person to mentally travel back in time to re-experience a past episode—or, so-called *autonoetic awareness* (Tulving, 1983)—and to be aware of “… the temporal dimension of their own and others' existence…” –referred to as *chronosthesia* (Tulving, 2002, p. 313).

A wide range of language-based paradigms (e.g., word lists, mental imagery tasks, navigation tasks, autobiographical memory questionnaires) have been used to investigate episodic memory in human adults (e.g., Tulving, 1972; Williams and Broadbent, 1986; Hassabis et al., 2007; Mullally et al., 2012). In most of these paradigms, participants are asked to describe the content of a memory and the subjective experience (i.e., type of awareness) associated with remembering this content (e.g., Levine et al., 2002; Buckner and Carroll, 2007).

There is no doubt that participants in such tasks are retrieving episodic memories. Nonetheless, researchers have little control over how participants have formed these memories or how often these memories have been retrieved (Pause et al., 2013). Recent studies have also shown a lack of inter-task relations thus calling into question the extent to which these different measures tap the same type of memory (e.g., Cheke and Clayton, 2013, 2015). In addition, relying exclusively on language-based tasks poses important challenges for testing episodic memory in non-verbal populations (e.g., pre-verbal children, non-human animals), and thus precludes making important comparisons across development and across species.

In order to overcome some of these limitations, there has recently been an increasing interest in developing non-language-based tasks grounded on the behavioral components of episodic memory. These tasks usually take the form of assessing the ability to remember *what* happened, *where*, and *when* (Tulving, 1972), and have been adapted from a depletion paradigm that was first developed for use with birds (Clayton and Dickinson, 1998). In their study, Clayton and Dickinson had scrub-jays (*Aphelocoma californica*) cache two types of food in different locations—preferred, but perishable, wax worms, and less-preferred, but non-perishable, peanuts. Importantly, the scrub jays could either recover the food after a short or long retention interval. At recovery, scrub-jays searched for worms after a short time had passed since caching, but switched to peanuts after a long time had elapsed since caching. Thus, birds successfully recalled the type of food they had cached (i.e., “what”), its location (i.e., “where”), and how long ago (i.e., “when”) they had cached it (Clayton and Dickinson, 1998). Because the paradigm did not directly assess the phenomenological components of the scrub-jays' memories, the authors concluded that scrub-jays had “episodic-like memories.”

Recent studies have shown that in “episodic-like” memory paradigms human adults also recall what, where, and when something happened (e.g., Pause et al., 2010; Plancher et al., 2010; Holland and Smulders, 2011; Easton et al., 2012; Cheke and Clayton, 2013; Mazurek et al., 2015; Craig et al., 2016). In these studies, participants are usually asked to recall, for example, in which room (e.g., Holland and Smulders, 2011; Craig et al., 2016) or quadrant of a computer screen (Pause et al., 2010) (i.e., “where”) and in which order (i.e., “when”) coins (e.g., Holland and Smulders, 2011; Craig et al., 2016) or visual stimuli (Pause et al., 2010) (i.e., “what”) were hidden or seen before. Crucially, adults' successful performance in these tasks has been interpreted as evidence that the what-where-when paradigms rely on episodic memory. However, Martin-Ordas et al. (2017) have suggested that there are at least two important differences between the studies with humans and Clayton and Dickinson (1998) depletion paradigm: (1) the definition of the “when” component, and (2) the behavioral criteria used to assess episodic memory.

In the studies with humans conducted thus far, “when” is defined as the “order” of events (henceforth “what-where-in which order” paradigms), whereas in the studies with the scrub-jays, it is defined as “how long ago” an event took place (henceforth “what-where-how long ago” paradigm). This difference in the definition of the “when” component is a particularly relevant issue because it has been argued that the “how long ago” component does not necessarily test *chronosthesia* which, as mentioned earlier, Tulving (2002) defined as a critical feature of episodic memory (e.g., McCormack, 2001; Roberts et al., 2008). For example, Roberts et al. (2008) suggested that in a what-where-how long ago paradigm “instead of remembering when an event happened within a framework of past time, animals are keeping track of how much time has elapsed since caching or encountering a particular food item at a particular place and are using elapsed time to indicate return to or avoidance of that location” (p. 113). Thus, even if successful performance in what-where-in which order tasks relies on episodic memory, the same might not be true for successful performance in the what-where-how long ago task.

As for the behavioral criterion used to assess episodic memory, Clayton and Dickinson (1998) measured scrub-jays' correct choices (i.e., choosing worms after the short retention interval, and peanuts after the long retention interval). In contrast, humans' episodic memories are usually measured by their verbal responses to the “what” (e.g., coins), “where” (e.g., in which room), and “when/in which order” (e.g., order in which the coins were hidden) questions (although see Pause et al., 2010 and Pause et al., 2013 for exceptions). Thus, in these studies, no measure of whether or not participants use duration to make choices (e.g., choose the preferred food after a short interval and the less preferred food after a long interval) was included- this being the crucial measure in the episodic-like memory paradigms used with non-human animals.

In order to address these two issues, Martin-Ordas et al. (2017) developed a what-where-how long ago depletion paradigm for children in which correct choices as well as responses to “what,” “where,” and “how long ago” questions were assessed. In two trials, 3-, 4-, and 5-year-olds were presented with a preferred food (i.e., popsicle) that was only edible after a short interval, and a less preferred food (i.e., raisins) that was edible after both short and long intervals. To make a successful choice, children had to remember what food item was hidden where as well as how much time had elapsed between the hiding of the two food items. Results showed that children chose their preferred food after the short intervals but, strikingly, did not select their less-preferred food after the long intervals. Consistent with previous findings, however, age-related changes in children's ability to remember “what” was hidden “where” were found. Nonetheless, children struggled at estimating the duration of the trials—a potential explanation for why they failed to make the correct critical choice in the depletion paradigm. However, a more controversial interpretation of Martin-Ordas et al.'s (2017) findings is that what-where-how long ago depletion paradigms do not necessarily rely on episodic memory.

One way to address this issue is to test human adults in the what-where-how long ago task previously used with preschool children. This is because adults not only have episodic memories but also have less difficulty at estimating temporal duration. In two experiments, we presented adult participants with a depletion task which involved choosing a food snack either after 3-min or 1-h. In Experiment 1 participants were asked to *imagine* the time between the hiding of the food items (a preferred but perishable grape popsicle and a less-preferred but non-perishable box of raisins) and choosing one of them. Successful performance would depend on participants' memory for what and where as well as on their ability to integrate temporal information into their decision-making process. In Experiment 2, participants *experienced* the time elapsed between the hiding of the food items and choosing one of them. Thus, Experiment 2 allowed us to assess whether adults would *remember and incorporate* temporal information to guide their choices in what-where-how long ago tasks. Participants' success would support the claim that depletion paradigms assess episodic thinking.

## Experiment 1: Questionnaire Version

We developed a questionnaire version of Martin-Ordas et al.'s (2017) procedure with children. On a screen in a lecture theater, participants were shown the setup used by Martin-Ordas et al. (2017) (i.e., an image of a table with three opaque boxes) and the images of two snacks: a preferred but perishable grape popsicle and a less-preferred but non-perishable box of raisins. Participants were asked to imagine that the two snacks were hidden under two of three boxes. Next, each participant was provided with a questionnaire in which they were asked to imagine choosing one of the three containers either after 3-min or 1-h. Correct responses (i.e., choosing the popsicle after 3-min and the raisins after 1-h) would indicate that adults are able to integrate “what,” “where,” and “when” information (i.e., hypothetical temporal distance between hiding the snacks and having to choose a container).

### Methods

#### Participants

An opportunistic sample of 84 University undergraduates was tested; 23 were excluded due to food preference (e.g., they did not like raisins, they liked raisins more than popsicles), resulting in a final sample of 61 (46 females; 15 males). All participants were predominantly White, and fluent in English. Participants were informed that participation was voluntary and that they could leave the lecture theater if they did not want to participate in the study.

#### Materials and Procedure

On a projector screen, we presented the images of a popsicle, a box of raisins, and a platform with three opaque cardboard boxes on top of it. Each participant was provided with a two-page questionnaire containing (1) a *food preference test* (page 1), (2) a *critical choice question*, and *memory check questions* (page 2).

1. *Food preference test*. The images of a popsicle and a box of raisins were shown to the participants and they were told “*Imagine that I have a popsicle and a box of raisins.”* Next participants were asked to look at page 1 of the questionnaire and answer the questions on it: (1) *Do you like popsicles? Yes/No*, (2) *Do you like raisins? Yes/No*, (3) *What do you like best: popsicles or raisins?* Participants were told not to share their answers with their classmates and they were asked to turn the paper over once they finished answering the questions.
2. *Critical choice question and memory-check questions*. Next, the image of a platform with three boxes on it was shown to the participants, and the Experimenter (E) said “Imagine that I am going to hide the popsicle under the right box, the raisins under the left box and the box in the middle remains empty.” Then, participants were asked to look at page 2 of the questionnaire and answer the questions: (1) *Imagine that in 1-h/3min you can have what is inside one of these boxes: which one would you choose? Left box/Middle box/Right box;* (2) *Do you remember which box has the popsicle? Left box/ Middle box/ Right box;* (3) *Do you remember which box has the raisins? Left box/ Middle box/ Right box*. Participants were again told not to share their answers with their classmates and they were asked to turn over the paper once they finished answering the questions.

#### Scoring and Analyses

##### Critical choice question

If participants selected the correct box, they received a score of 1, whereas if they selected an incorrect box, they received a score of 0. As in the depletion paradigms, choosing the box that contained the popsicle in the 3-min trials was scored as “correct” because it is the preferred food and is still edible, whereas choosing the empty box or the box containing the raisins was scored as “incorrect.” In contrast, in the 1-h trials, choosing the box containing the raisins was considered “correct,” and choosing the empty box or the box containing the popsicle (which would have melted and thus no longer be edible) was considered “incorrect.”

##### Memory-check questions (i.e., “*do you remember where the popsicle is*? *do you remember where the raisins are*?”)

Participants received a score of 1 if they answered that the box on the right contained the popsicle, and that the box on the left contained raisins. Any other response was scored as 0.

##### Analyses

We used Pearson chi-square tests to analyze performance in the critical choice question. We used binomial tests to assess whether participants were above chance in the critical choice question and memory check questions (chance = 33%). All statistical tests were exact two-tailed, and results were considered significant if *p* < 0.05.

### Results

#### Critical Choice Question

**Figure 1:**
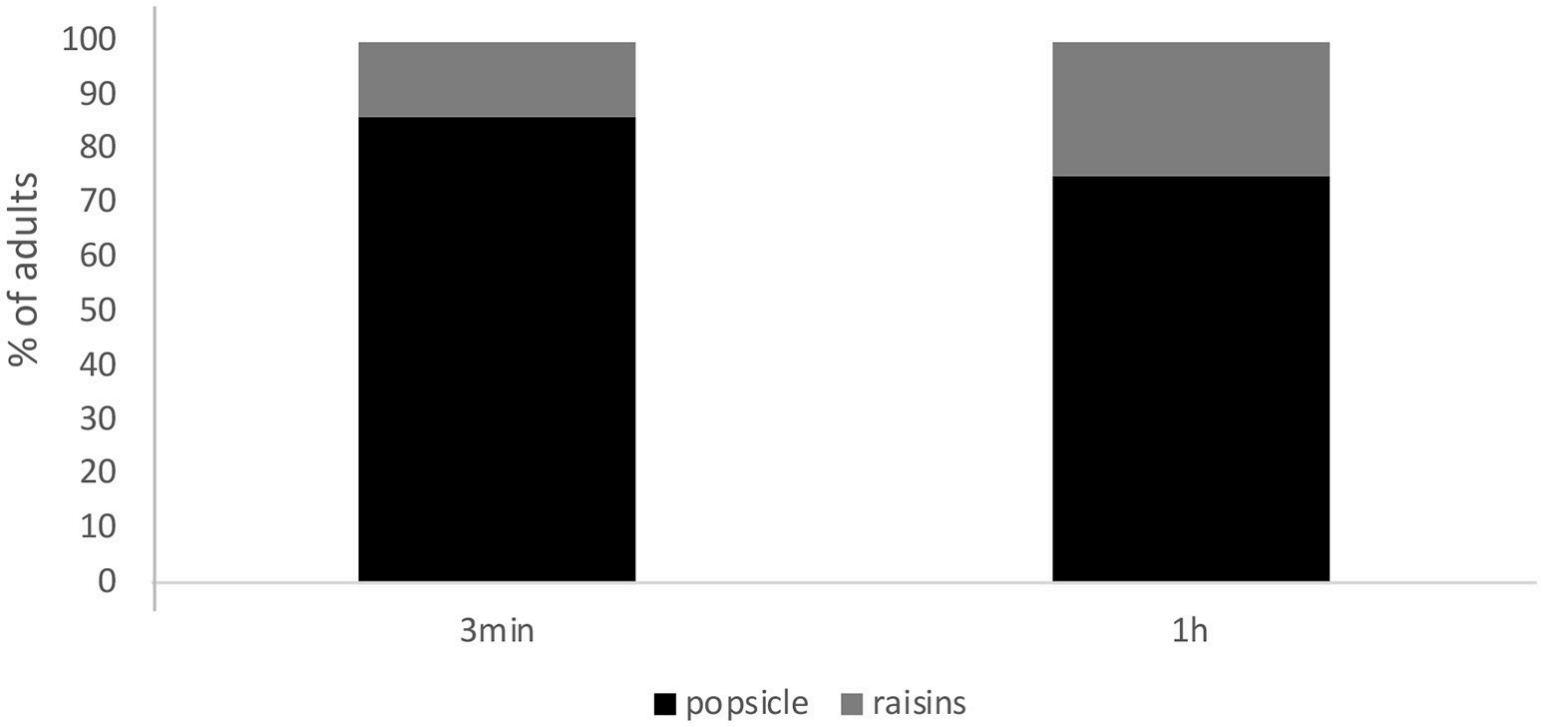
Percentage of adults who chose the box containing the popsicle or the box containing the raisins in the critical choice question grouped as a function of retention interval (RI) and trial type. Note that choosing the popsicle in the 3-min trial was considered correct and choosing the raisins in the 1-h trial was considered correct.

Participants performed significantly better in the 3-min trial compared to the 1-h trial (χ^2^ = 15.74, *df* = 1, *p* < 0.001). Binomial tests indicated that participants chose the box containing the popsicle significantly above chance in the 3-min trial (*p* < 0.001) but failed to choose the box containing the raisins significantly above chance in the 1-h trial (*p* = 0.87). In fact, 76% of the adult participants chose the box containing the popsicle after 1-h (*p* < 0.001) (see Figure 1).

#### Memory-Check Questions (“What Is Where”)

Participants' responses to the “what is where” question was significantly above chance in both the 3-min (Binomial test: *p* < 0.001; 97% of the participants answered this question correctly) and 1-h trials (Binomial test: *p* < 0.001; 91% of the participants answered this question correctly), and did not differ as a function of trial type (χ^2^ = 1.42, *df* = 1, *p* = 0.285). In other words, participants' memory about where the popsicle and raisins were hidden was not the limiting factor in their performance.

### Discussion

We developed a questionnaire version of the what-where-how long-ago paradigm previously used with non-human animals and preschool children for use with adults. Strikingly, participants chose the preferred and perishable food (i.e., popsicle) *both* after 3-min and 1-h. Participants' responses to the memory-check questions revealed that failure to remember what was hidden where cannot explain our results. One could argue that participants' choices of their preferred food after 1-h could be due to participants' inability to integrate the temporal information with their knowledge about the perishability of the food items. However, it is also possible that temporal information was not salient enough in the current task. This is because participants were provided with the duration of the trials in the critical choice question, but did not actually experience the time between the hiding of the food items and choosing a container. This is an important difference between our method and previous studies using this paradigm. Another possibility is that participants were not sufficiently motivated by the food “rewards”—note that, contrary to the studies with non-human animals and children, our participants were not presented with real rewards but, rather, photographs of them.

In order to control for these alternative explanations, in Experiment 2, participants were presented with the same procedure developed by Martin-Ordas et al. (2017) for use with children. In this what-where-how long ago task participants experienced the time between hiding two real food rewards and choosing one of the containers. As in Experiment 1, we predicted that if this task draws on episodic memory, participants will successfully choose their preferred food snack after 3-min and their less preferred food after 1-h.

## Experiment 2: Lab Version

Following Martin-Ordas et al. (2017), we presented adults with two trials in which they witnessed an Experimenter hiding two snacks—a preferred, but perishable grape popsicle, and a less-preferred, but non-perishable box of raisins- in two of three locations on a platform. Participants were asked to choose from one of the three locations (i.e., critical choice question) after a 3-min or 1-h retention interval (RI) and to answer a series of memory questions about “what” we hid, “where,” and “how long ago” we hid it (i.e., memory-check questions). Importantly after 3-min, the popsicle was still edible, whereas after 1-h it was not (i.e., it had melted).

Crucially the current paradigm also allowed us to investigate participants' correct choices—this measure was the equivalent of scrub jays choosing worms or peanuts in Clayton and Dickinson (1998)—as well as participants' recollection of behavioral components of episodic memory—this measure being similar to those assessed in previous studies with humans. In addition, presenting participants with two trials allowed us to assess how they respond to an “unexpected” question about a past event or, what has been termed “incidental encoding” (Zentall et al., 2001, 2008). We explored this last issue by analyzing participants' responses in Trial 1—when they were unaware of what the task would involve–and Trial 2—when they knew what the task would entail. We decided to include this manipulation because it has been argued that a feature of episodic memory is that recollection can occur when encoding is incidental and memory assessment is unexpected (Zentall et al., 2001, 2008). Importantly, recent studies have shown that manipulating the level of intentionality during the encoding phase (intentional encoding vs. incidental encoding) affects recollection for “what,” “where,” and “in which order” something happened (e.g., Holland and Smulders, 2011; Craig et al., 2016). Finally, we were also interested in investigating the relation between the different measures of episodic memory used in the present study—correct choices in the depletion paradigm and recollection of the behavioral components of episodic memory. A positive relation would support the claim that both measures rely on the same type of memory (i.e., episodic memory).

We hypothesized that if participants remember what, where, and how long ago in an integrated manner (e.g., Clayton and Dickinson, 1998; Clayton et al., 2003), they would choose the popsicle (preferred food) after 3-min has passed and the raisins (less-preferred food) after 1-h has passed. Since the intentionality at encoding has been shown to affect recollection (e.g., Holland and Smulders, 2011), we expected participants to perform better in the second trial compared to the first—both in terms of correct choices and responses to the memory check questions. In particular, we predicted that those participants who received the 1-h RI in Trial 1 (i.e., experienced the melted popsicle) should perform better on the 1-h RI in Trial 2, than those who received the 3-min RI in Trial 1. Finally, if our measures (i.e., correct choices, responses to the memory check questions) tap the same type of memory (i.e., episodic memory), then scores on these measures should be positively correlated. Although previous studies have investigated the relation between what-where-in which order, free recall, and source memory tasks (e.g., Cheke and Clayton, 2013), our study is the first to investigate the relation between the responses used in episodic-like memory tasks in animals and the responses used in episodic-like memory tasks in humans.

### Methods

#### Participants

Thirty-five University undergraduates were recruited; 11 were excluded due to food preference (e.g., they did not like raisins, they liked raisins more than popsicles) or failure to attend both sessions, resulting in a final sample of 24 (15 females; 9 males). All participants were predominantly White, middle class, and fluent in English. The research was approved by the Office of Research Ethics and Integrity at the University of Ottawa. Participants provided written informed consent.

#### Materials and Procedure

**Figure 2:**
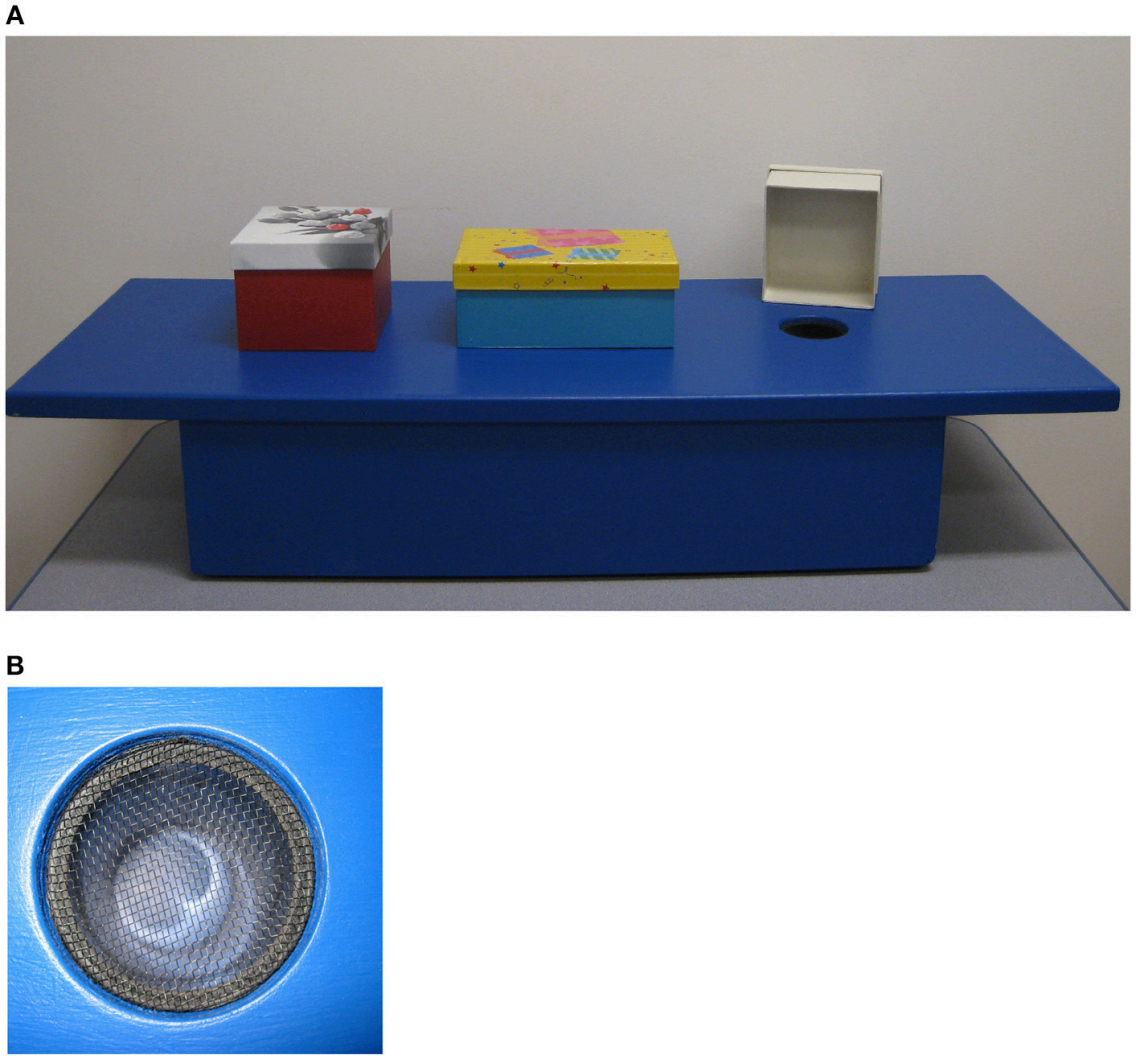
Apparatus used in the present study. The photo depicts **(A)** the three cardboard boxes and the wooden platform and **(B)** a detail of one of three holes and plastic netting.

We used the exact same materials and procedure as in Martin-Ordas et al. (2017). There were three different cardboard boxes (~12 cm wide × 19 cm long × 8.8 cm high each) and a wooden platform (91 cm long × 75.5 cm wide) in which three holes (5 cm diameter) were drilled and then covered with a plastic netting (see Figure 2). This plastic netting allowed liquid (from the melting popsicle) to pass through and collect inside a cup that was hidden under the platform. The experiment took place in two rooms: Room 1, where the hiding event took place, and Room 2, where the participants waited either 3-min or 1-h—depending on the type of trial.

Participants received two trials separated by five to seven days and each trial consisted of five main events: (1) food preference test, (2) hiding event, (3) critical choice question, (4) memory check questions and, (5) “how long ago” question.

1. *Food preference test*. E and participant sat facing each other. E placed a box of raisins (4.6 cm long × 3.4 cm wide × 1.7 cm high) and a popsicle (3 cm long × 2.5 cm wide × 1.5 cm high) on two small dishes and asked participants “*Which one of these two snacks do you like best: popsicles or raisins*?” Note that at this point participants did not receive either food item. Next, E proceeded with the hiding event.

2. *Hiding event*. E placed the three cardboard boxes on the platform. For each of the two snacks E said: “*Look what I have here! I am going to put it here*.” E then placed the popsicle under one of the three boxes, the raisins under another one and the third box remained empty. Hiding locations and box locations were counterbalanced within and across participants. The rationale for having an empty box was to control for participants remembering which boxes had food under them. However, participants never chose the empty box in Trial 1 or in Trial 2.

There were two types of trials defined by the length of time/RI that elapsed between hiding the food items and allowing participants to choose one of the boxes (i.e., critical choice): 3-min and 1-h. On the 3-min trials, the popsicle and raisins were both available (i.e., edible), whereas on the 1-hour trial the popsicle melted and only the raisins were edible. Fifty percent of the participants received the 3-min trial first followed by either the 3-min trial or 1-h trial. The other 50% received the 1-h trial first followed by either the 3-min or 1-h trial. Thus, the combination of trial type and order of presentation yielded 4 experimental conditions: 1-h (first) trial and 1-h (second) trial; 1-h trial and 3-min trial; 3-min and 3-min trial; 3-min and 1-h trial. Participants were randomly assigned to each of the conditions. During the RIs, participants went to Room 2 and were engaged in unrelated activities (e.g., reading). Importantly, before leaving Room 1, E clearly stated “*the door is going to be locked so no one can go inside the room while we are not there*.”

3. *Critical choice question*. After 3-min or 1-h, E and participant returned to Room 1 and E asked the participant the critical choice question, “*Now you can have what is inside one of these boxes. Which one are you going to choose*?” Our critical choice question is analogous to scrub jays being allowed to retrieve a particular food (e.g., peanuts or wax worms) after a predetermined RI. In the 1-h trials, and once the box was uncovered and participants had answered the memory-check and how long ago questions, E asked participants “*What happened to the popsicle*?” All participants stated that the popsicle had melted, thus confirming that they understood the melting process.

4. *Memory-check questions*. E asked three memory-check questions to assess whether participants remembered “what” (“*Do you remember what I put under the boxes*?”), “where” (“*Do you remember which boxes have something under them*?”) and “what is where” (“*Do you remember where the popsicle is*? *Do you remember where the raisins are*?”). These questions are similar to those used to measure episodic memory in the studies with adults. Half of the participants were asked the critical choice question first and the memory-check questions second, whereas for the other half this order was reversed. However, only after participants decided on the location/box they wanted to uncover, and answered the memory check questions, were they shown the content of their chosen box.

5. *How long ago question*. We always asked this question at the end of the trial, and worded it as follows: “*Do you remember when we were in the other room (i.e., Room 2)? Did it feel like the time that it takes to brush your teeth, or like the time that it takes to make dinner and then eat it with your family?”* Similar to the experiment with the children (Martin-Ordas et al., 2017), E showed participants two pictures while presenting these two different options; one depicted a person brushing her teeth, and the other depicted a woman cooking with her family and then having dinner. To provide participants with a graphic representation of the duration of the actions, two lines were drawn under each of the two pictures: a short line for “brushing teeth,” and a longer line for “making and eating dinner.” The rationale behind the “how long ago” question was to assess whether incorrect responses on the critical choice question (e.g., choosing the popsicle after a 1-h RI) were due to difficulties estimating the amount of time/duration of the RIs.

#### Scoring and Analyses

Trials were video-recorded and participants' choices were scored as a function of which box they pointed to first (correct box = 1; incorrect box = 0).

##### Critical choice question

Similar to scoring used in previous studies using the depletion paradigm, choosing the box hiding the popsicle in the 3-min trials was scored as “correct” because it is the preferred food and is still edible, whereas choosing the empty box or the box hiding the raisins was scored as “incorrect.” In contrast, in the 1-h trials, choosing the box hiding the raisins was considered “correct,” and choosing the empty box or the box hiding the popsicle (which had melted and was no longer edible) was considered “incorrect.”

##### Memory-check questions (“what,” “where,” and “what is where”)

Participants received a score of 1 for the “what” question (i.e., “*Do you remember what I put under the boxes*?”) if they responded with both “*popsicle*” and “*raisins*.” Any other response was scored as 0. Participants received a score of 1 for the “where” question (i.e., “*Do you remember which boxes have something under them*?”) if they pointed at the two boxes that contained the food items. Any other response was scored as 0. For the binding of “what is where” (i.e., “*Do you remember where the popsicle is*? *Do you remember where the raisins are*?”), participants received a score of 1 if they pointed at the box containing the popsicle, and at the box containing the raisins. Any other response was scored as 0.

##### “How long ago” question

For the “how long ago” question (i.e., “*Do you remember when we were in the other room? Did it feel like the time that it takes to brush your teeth, or like the time that it takes to make dinner and then eat it with your family?”)*, participants received a score of 1 if they answered “*brushing teeth*” after the 3-min trial, and “*making and eating dinner*” after the 1-h trial.

##### Analyses

We used Pearson chi-square tests to analyze performance in the critical choice question in Trial 1, and also performance on the critical choice question in Trial 2 as a function of what type of trial participants received first. We used binomial tests to assess whether participants were above chance in the critical choice question, memory check questions (chance = 33%), and “how long ago” question (chance = 50%). However, because participants' performance was at ceiling in the memory check and “how long ago” questions, correlations between the different measures could not be calculated. Thus, we used a Friedman's test to analyze whether there were differences between the different measures. To do so, proportion scores (i.e., participants' overall success in both trials) were created for the three variables. All statistical tests were exact two-tailed, and results were considered significant if *p* < 0.05.

### Results

#### Critical Choice Question

##### Performance in Trial 1

Participants performed significantly better in the 3-min trial than in the 1-h trial (χ^2^ = 10.66, *df* = 1, *p* = 0.001). Binomial tests indicated that participants chose the box hiding the popsicle significantly above chance in the 3-min trial (*p* < 0.001) but failed to choose the box hiding the raisins significantly above chance in the 1-h trial (*p* = 0.37). In fact, 83% of the adult participants chose the box hiding the popsicle after 1-h (*p* < 0.001).

##### Performance in Trial 2 as a function of Trial 1

**Figure 3:**
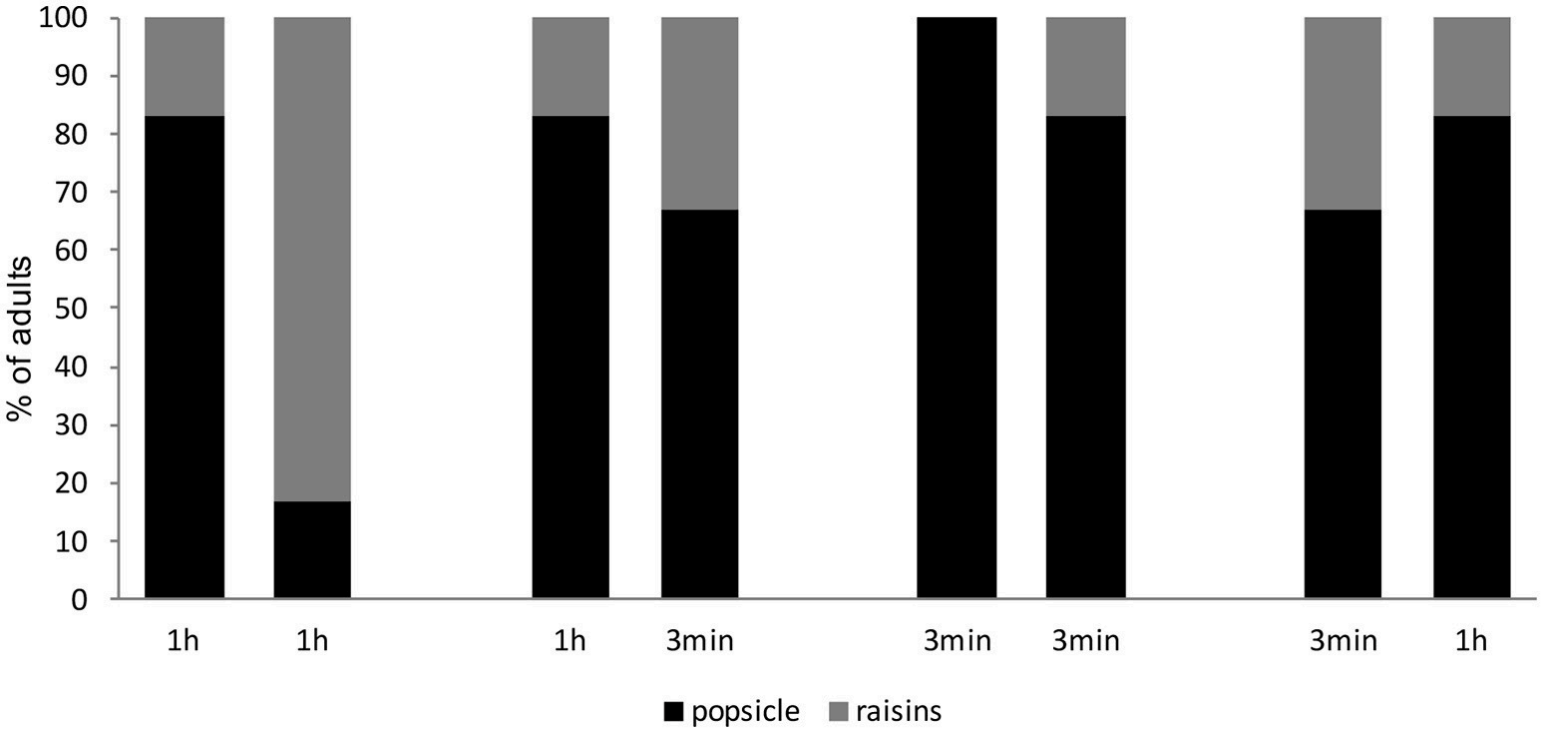
Percentage of adults who chose the box hiding the popsicle or the box hiding the raisins in the critical choice question grouped as a function of RI and trial type. Note that choosing the popsicle in the 3-min trial was considered correct and choosing the raisins in the 1-h trial was considered correct.

To investigate the effect of previous experience, we analyzed performance in Trial 2 as a function of the trial participants received first. Performance in the second 1-h trial was superior for those participants who received the 1-h trial first as compared to those who received the 3-min trial first (χ^2^ = 5.33, *df* = 1, *p* = 0.021). However, performance in the second 3-min trial was not affected by whether participants received a 3-min or 1-h RI in Trial 1 (χ^2^ = 0.444, *df* = 1, *p* = 0.505). Together, these results show that participants' choices after the 1-h RI in Trial 2 were significantly affected by which trial they received first (see Figure 3).

Further analyses revealed that adults in the 1-h RI performed significantly above chance when they received the 1-h RI in Trial 1 (binomial test, *p* = 0.017) but not when they received the 3-min RI first (binomial test, *p* = 0.35). Those participants who received the 3-min RI in Trial 2 performed significantly above chance when they received the 3-min RI in Trial 1 (binomial test, *p* = 0.017), but those who received the 1-h trial in Trial 1 did not (binomial test, *p* = 0.097).

#### Memory-Check Questions (“What,” “Where,” “What Is Where”)

##### Performance in Trial 1

Participants' memory for “what,” “where,” and “what is where” did not differ as a function of trial length. In fact, all participants correctly responded to these questions in the 3-min and 1-h trials.

##### Performance in Trial 2

As in Trial 1, participants' performance on the memory-check questions did not differ between the 3-min RI and the 1-h RI. As before, all participants responded to the three questions correctly.

#### How Long Ago Question

##### Performance in Trial 1

Ninety-six percent of participants correctly estimated the duration of the 1-h RI and 100% did so for the duration of the 3-min RI.

##### Performance in Trial 2

All participants correctly estimated the duration of the trial for both the 3-min and 1-h RIs.

#### Relation Between the Critical Choice, Memory-Check, and “How Long Ago” Questions

Because participants' performance was at ceiling in the memory-check and “how long ago” questions, correlations could not be calculated. Thus, we analyzed whether there were differences between the overall performance in the critical choice question (i.e., combined score on Trials 1 and 2), overall performance in the “what- where-how long ago” questions (i.e., combined score for these three questions on Trials 1 and 2) and overall performance in the binding question “what is where” (i.e., combined score on Trials 1 and 2). Friedman test of differences between overall scores on the critical choice question, “what-where-how long ago” questions, and the binding of “what is where” was calculated and rendered a χ^2^ = 51.21, which was significant (*p* < 0.001, *n* = 24). *Post-hoc* Wilcoxon tests showed that participants performed worse in the critical choice question compared to the “what-where-how long ago” questions (*Z* = −3.879, *n* = 17, *p* < 0.001), and the “what is where” question (*Z* = −4.001, *n* = 18, *p* < 0.001).

## Discussion

We adapted the what-where-how long ago paradigm previously used with non-human animals and preschool children for use with adults. In Trial 1, participants chose the preferred and perishable food (i.e., popsicle) after the short RI but did not choose the less preferred, non-perishable food (i.e., raisins) after the long RI. However, experiencing the melted popsicle in Trial l improved participants' performance in Trial 2. We also assessed recollection for “what,” “where,” “what is where,” and “how long ago” and found that adults' performance was at ceiling on these measures in both trials. Finally, we analyzed whether there were differences in difficulty between our measures and found that participants performed significantly worse in the critical choice question than in the “what-where-how long ago” and “what is where” questions.

## General Discussion

In two experiments we presented adults with a what-where-how long ago task. Strikingly, participants struggled to adapt their food choices to the length of the trial. This was irrespective of whether they were asked to imagine (Experiment 1) or actually experienced (Experiment 2) the time elapsed between the hiding of the food rewards and choosing one of the containers. Our results show that memory for the contents of the boxes cannot account for participants' failures in the critical choice questions. Rather, Experiment 2 highlights the role that previous experience might play in depletion paradigms.

### Critical Choice Questions

Participants' performance on the critical choice questions in Experiment 1 and Trial 1 of Experiment 2 was rather unexpected. Although they chose their preferred food after the short RI, they did not correctly choose their less preferred food after the long RI. One could argue that participants may not have been motivated by the food rewards because they were neither particularly hungry nor thirsty. Yet, our observations of participants' reactions in Experiment 2 were quite the opposite—that is, participants expressed disappointment upon seeing that the popsicle had melted. Moreover, when they successfully obtained the reward—either the popsicle or the raisins—participants consumed it immediately after the experimenter gave it to them. As such, we do not think that lack of motivation can account for our findings.

We can also rule out the possibility that participants lacked “semantic” knowledge about “melting” given that adults understand the transformation of certain substances (e.g., ice melts with time). This understanding was also confirmed by their responses to the “*What happened to the popsicle?*” question in Experiment 2 (i.e., all adults stated that it had melted). Importantly, in Experiment 2 we found quite a different pattern of results on the critical choice question for the second 1-h trial. More specifically, in the 1 hour-1 hour condition adults' performance on the critical choice question of Trial 2 significantly improved. These findings suggest that participants correctly chose their less preferred food (i.e., raisins) *only* when they had previously experienced the melted popsicle.

What are the exact mechanisms that can account for participants' improvement on the critical choice question of the second 1-h trial in Experiment 2? One possibility is that those participants who experienced the melted popsicle in Trial 1 avoided choosing the popsicle in Trial 2—regardless of the duration of the trial. This seems unlikely though given that 67% of the participants still chose the popsicle in the 3-min RI in Trial 2, after experiencing its melting (i.e., 1-h RI) in Trial 1. It also seems unlikely that this improvement was due to a change in participants' preferences in the second trial because participants who received the 3-min trial first chose the popsicle in the second trial independently of its duration.

More plausible is that participants' experience in Trial 1 subsequently shifted their attention in Trial 2 to the relation (i.e., binding) between the elements of the problem (Clayton et al., 2003); that is, to make a correct choice, participants not only had to remember the contextual information (i.e., “what,” “where,” “what is where”) and the temporal information (i.e., “how long ago”), but also integrate them—“how long ago a particular food item was placed where.” In fact, whereas one could argue that participants' responses to the critical choice question in Trial 1 could be explained by simply choosing their preferred food—independently of the duration of the trial- integrating the temporal information with the contextual information can conceivably explain their performance in Trial 2. In this sense, our results do not differ from those reported with the scrub-jays (e.g., Clayton and Dickinson, 1998). In Clayton and Dickinson's experiment, scrub-jays experienced four pre-training trials in which they had the opportunity to learn that worms degrade and become inedible after a long time has passed between caching and recovery. Thus, it is conceivable that becoming aware of how the passing of time affects the edibility of the food items is crucial to succeed in a what-where-how long ago task for both humans and non-human animals. An interesting direction for future studies would be to address this issue by directly telling participants how long it takes a popsicle to melt. If becoming aware of the temporal information facilitates performance in the depletion paradigms, participants should succeed in this version of the task. Relatedly, showing that participants' performance generalizes to other kinds of “depletion” paradigms that do not use food as stimuli is also important. For example, one could imagine developing a task in which there is an electronic device (e.g., i-Pad) that plays a preferred game/show but that has a battery that runs out quickly vs. a device that plays a less preferred game/show but has a longer-lasting battery. If participants do indeed have difficulty using duration information in their decision-making process (as we have argued), they should fail to choose the “longer-lasting” device/less-preferred game after the long delay—just as participants in our experiments failed to choose the less-preferred raisins. This pattern of results would suggest that our findings are not specific to one particular domain of reasoning, such as “food.”

### Memory-Check and How Long Ago Questions

Consistent with results from previous studies (e.g., Plancher et al., 2010; Holland and Smulders, 2011; Craig et al., 2016), adults in our study accurately remembered “what,” “where,” and “what was hidden where” and, in Experiment 2, also correctly estimated the length of both Trials 1 and 2—a novel feature of our study. Consequently, failing to recall this contextual information cannot account for participants' poor performance in the first 1-h trial. Rather, as mentioned earlier, adults' difficulty appeared to be rooted in their inability to precisely *use* duration information. Indeed, although adults accurately judged trial duration, they did not appear to integrate this information to then allow them to decide that, after 1-h, the popsicle will have melted. Thus, *using* duration information when deciding which box to choose appears to be a key to success in the current what-where-how long ago task.

### Incidental Encoding and the Role of Previous Experience

In Experiment 2, participants' improved performance in Trial 2 is also consistent with arguments that the memory processes involved in a first encounter—or “trial,” in the context of our study—of an event differ from those involved in subsequent encounters (or “trials”) (Zentall et al., 2001, 2008; Plancher et al., 2010). Most notably, Zentall et al. (2001, 2008) argued that deliberate encoding (e.g., use of training phases) helps organisms develop expectations of future rewards. The development of such expectations favors the storing of this information as semantic rather than episodic memories. Thus, in the context of our tasks, this suggests that when participants do not know what they are going to be asked, episodic memory is not sufficient to succeed in the critical choice question (i.e., Experiment 1 and Trial 1 of Experiment 2); however, when they do know (i.e., Trial 2), participants might integrate the spatio-temporal information with the non-episodic information (e.g., semantic facts) to make the correct choice (e.g., “choose the non-perishable food after 1-h”).

These results are not only consistent with adults' performance in previous what-where-in which order tasks (Holland and Smulders, 2011; Plancher et al., 2012; Craig et al., 2016) but also with preschoolers' performance in the what-where-how long ago task (Martin-Ordas et al., 2017). Specifically, children's successful estimation of “how long ago” the hiding event took place was related to successful performance in the critical choice question. Crucially, this effect was only true for Trial 2 - that is, once children knew what the task entailed. Martin-Ordas et al. (2017) argued that children might not spontaneously incorporate the duration of the trial into their decisions. This finding is consistent with the results of Experiment 2: Once adults experienced the melted popsicle, they were able to take into account the duration of the trial in order to make their choices.

### Comparisons Between Our Measures

Experiment 2 also allowed us to investigate the degree of relation between the different measures used in depletion paradigms. Adults' performance differed between the critical choice question, the what-where-how long ago questions and the “what is where” question. Participants' better performance in the “memory check” questions compared to the critical choice question also suggests that these measures might tap different memory systems. In fact, previous studies addressing the relation between different measures of episodic memory in adults have also reported such differences (e.g., Plancher et al., 2010; Easton et al., 2012; Cheke and Clayton, 2013; Pause et al., 2013). For example, Easton et al. (2012) found that whereas performance in a “what-where-in which context” task required recollection of the past event (i.e., episodic memory), performance in a “what-where-in which order” task did not. This finding led the authors to conclude that tasks that rely on temporal information might be susceptible to non-episodic strategies.

Although it is true that methodological differences could account for differences in the results across different studies, our task highlights the need to gain better consensus about the “when” component that is measured in episodic memory tests. Because this aspect has been tested in a variety of ways in both the human and animal cognition literatures, it is difficult to compare performance on this measure across studies. For example, whereas time of day (Roberts et al., 2008) or duration (e.g., Clayton and Dickinson, 1998) have been the main temporal markers used in the animal research, order of events (e.g., Cheke and Clayton, 2013; Mazurek et al., 2015; Craig et al., 2016) has been the main temporal marker used in previous “episodic-like” memory tasks with adult humans. Because these different temporal markers might require the involvement of different memory systems—as the current and previous studies suggest, comparing performance across studies is difficult. As such, an important goal for future research and theorizing is to more consistently operationalize the temporal component of the episodic memory system across studies. This is especially important when trying to validate methodologies previously used in the animal literature.

## Conclusion

Our episodic-like memory depletion paradigms showed that adult humans successfully took into account retention interval when deciding whether to choose a non-perishable or perishable food—but only *after* having experienced the event once before (i.e., 1-h RI in Trial 1 of Experiment 2). Consistent with previous findings, our results also showed that participants successfully remember episodic components of an event (e.g., “what,” “where,” “what is where”) and also, a new aspect of our task (Experiment 2), “how long ago” a particular event happened. These findings, therefore, suggest that recalling what-where-how long ago and deciding which food item to choose might rely on different memory systems.

## Author Contributions

GM-O and CA designed the experiments. GM-O collected the data. GM-O and CA analyzed the data and wrote the manuscript.

### Conflict of Interest Statement

The authors declare that the research was conducted in the absence of any commercial or financial relationships that could be construed as a potential conflict of interest.
